# Cell immunity to SARS-CoV-2 after natural infection and/or different vaccination regimens

**DOI:** 10.3389/fcimb.2024.1370859

**Published:** 2024-03-20

**Authors:** Esther Culebras, Mercedes Martínez, Consuelo Novella, Jose Manuel León, Esther Marcos, Alberto Delgado-Iribarren, Esther Ríos

**Affiliations:** ^1^Servicio de Microbiología Clínica, Instituto Medicina Laboratorio (IML), Fundación para la Investigación Biomédica del Hospital Clínico San Carlos (IdISSC), Hospital Clínico San Carlos, Madrid, Spain; ^2^Departamento de Medicina, Facultad de Medicina, Universidad Complutense, Madrid, Spain; ^3^Sala de extracciones, IML, Hospital Clínico San Carlos, Madrid, Spain

**Keywords:** SARS-CoV-2, vaccine regimens, cellular immunity, humoral immunity, Astrazeneca, Pfizer-BioNTech, Moderna

## Abstract

**Background:**

The aim of the study was to evaluate the humoral and cellular immunity after SARS-CoV-2 infection and/or vaccination according to the type of vaccine, number of doses and combination of vaccines.

**Methods:**

Volunteer subjects were sampled between September 2021 and July 2022 in Hospital Clínico San Carlos, Madrid (Spain). Participants had different immunological status against SARS-CoV-2: vaccinated and unvaccinated, with or without previous COVID-19 infection, including healthy and immunocompromised individuals. Determination of IgG against the spike protein S1 subunit receptor-binding domain (RBD) was performed by chemiluminescence microparticle immunoassay (CMIA) using the Architect i10000sr platform (Abbott). The SARS-CoV-2-specific T-cell responses were assessed by quantification of interferon gamma release using QuantiFERON SARS-CoV-2 assay (Qiagen).

**Results:**

A total of 181 samples were collected, 170 were from vaccinated individuals and 11 from unvaccinated. Among the participants, 41 were aware of having previously been infected by SARS-CoV-2. Vaccinated people received one or two doses of the following vaccines against SARS-CoV-2: ChAdOx1-S (University of Oxford—AstraZeneca) (AZ) and/orBNT162b2 (Pfizer—BioNTech)(PZ). Subjects immunized with a third-booster dose received PZ or mRNA-1273 (Moderna—NIAID)(MD) vaccines. All vaccinees developed a positive humoral response (>7.1 BAU/ml), but the cellular response varied depending on the vaccination regimen. Only AZ/PZ combination and 3 doses of vaccination elicited a positive cellular response (median concentration of IFN- γ > 0.3 IU/ml). Regarding a two-dose vaccination regimen, AZ/PZ combination induced the highest humoral and cellular immunity. A booster with mRNA vaccine resulted in increases in median levels of IgG-Spike antibodies and IFN-γ as compared to those of two-dose of any vaccine. Humoral and cellular immunity levels were significantly higher in participants with previous infection compared to those without infection.

**Conclusion:**

Heterologous vaccination (AZ/PZ) elicited the strongest immunity among the two-dose vaccination regimens. The immunity offered by the third-booster dose of SARS-CoV-2 vaccine depends not only on the type of vaccine administered but also on previous doses and prior infection. Previous exposure to SARS-CoV-2 antigens by infection strongly affect immunity of vaccinated individuals.

## 1 Introduction

Since severe acute respiratory syndrome coronavirus 2 (SARS-CoV-2) emerged in Wuhan (China) in December 2019, the virus has spread rapidly, causing the coronavirus disease 2019 (COVID-19) pandemic (Meo et al., 2021). Several vaccines have been developed, successfully protecting against symptomatic COVID-19 cases and deaths. Among the vaccines available in Spain, two of themBNT162b2 (Pfizer—BioNTech) (PZ) and mRNA-1273 (Moderna—NIAID) (MD), are messenger RNA (mRNA) vaccines. Other vaccines, such as ChAdOx1-S (University of Oxford—AstraZeneca) (AZ), is based on the “Chimpanzee” adenovirus vector (Gobierno de España, 2024).

In response to SARS-CoV-2 infection, humans produce specific antibodies, CD4^+^ and CD8^+^ T cells. SARS-CoV-2-specific antibodies are directed against the spike protein (S) and nucleocapsid (N). Special roles are played by neutralizing antibodies against the S1 subunit on the receptor-binding domain (RBD) that binds to angiotensin-converting enzyme 2 (ACE2) sites, thereby facilitating endocytosis, viral entry into host cells (Tretyn et al., 2021). In case of the T cells (including CD4^+^ T and CD8^+^ T cells) recognize, in addition to the nucleocapsid and spike protein, membrane proteins (M) of the virus and are present in most COVID-19 patients (Sette and Crotty, 2021). It is known that while antibodies, produced by B cells in response to viral infection, provide a first line of defense against subsequent exposures, SARS-CoV-2 -specific-T cells (including CD4^+^ and CD8^+^ T cells) could limit disease severity, reduce its duration and drive rapid recovery (Kedzierska and Thomas, 2022). During natural infection, CD8^+^ T cells play an important complementary role to contain the infection through their ability to eliminate already infected cells, while CD4^+^ helper T cells, amongst other functions, provide signals that support the development of antibody responses (Castro Dopico et al., 2022). Therefore, a combined humoral and cell-mediated response are required for an optimal immunity to SARS-CoV-2 infection (Goletti et al., 2021).

In line with natural infection, SARS-CoV-2 vaccination has also been shown to induce robust humoral and T-cell responses (Goletti et al., 2021; Goel et al., 2021; Ben Ahmed et al., 2022; Zhang Z. et al., 2022). Vaccine-induced immunity remains effective at preventing severe disease, hospitalization, and death, even at later time points when antibody levels may have declined (Griffin et al., 2021; Thomas et al., 2021).

Hence, our purpose was to evaluate the humoral and cellular immunity after SARS-CoV-2 infection and/or vaccination according to the type of vaccine, number of doses and combination of vaccines.

## 2 Materials and methods

### 2.1 Participant recruitment

The present study is part of two projects in which the effects and immunity of several vaccines against SARS-CoV-2 were evaluated. These projects were approved by the Ethics Committee of Hospital Clínico San Carlos, Madrid (Spain) (References: 21/071-E and 21/193-E). All research was conducted according to the Declaration of Helsinki principles.

Blood samples were taken from volunteer subjects with different characteristics: COVID-19 vaccinated and unvaccinated, with or without previous COVID-19 infection, including healthy and immunocompromised individuals. The participants were recruited among the health workers of the Hospital Clínico San Carlos (HCSC) and through the HCSC retirees’ association. Other individuals came from different public administrations, mainly from the Spanish Civil Guard (Guardia Civil) and teachers of different levels (primary, secondary and University).

The enrolled individuals provided written informed consent for the collection of samples and subsequent analysis. Besides, the subjects completed a questionnaire indicating: sex, age, vaccination status, type and number of vaccines received and if they had previously been infected by SARS-CoV-2. Participants were sampled between September 2021 and July 2022 in Hospital Clínico San Carlos, Madrid (Spain). Whole blood samples were collected directly into lithium heparin blood tubes for later transfer to the assay tubes.

Participants were designated as previously infected for SARS-CoV-2 based on a positive PCR and/or serology (positive or negative results for anti-N antibodies with values ≥ 7.1 BAU/ml) at any time or according to self-reported status from the questionnaire.

### 2.2 Samples collection

Blood donations were obtained at different time points depending on the type of vaccine, for AZ from 2 to 10 months and for PZ from 6 to 12 months after receiving the second dose, respectively. In case of immunization with a third dose, blood samples were collected from 1 to 7 months after vaccination.

The specific immune response against SARS-CoV-2 antigens was evaluated according to the previous infection, type of vaccine, number of doses and combination of vaccines.

### 2.3 Determination of IgG antibodies against SARS-CoV-2

Determination of IgG against the SARS-CoV-2 spike protein S1 subunit receptor-binding domain (RBD) was performed using the chemiluminescence microparticle immunoassay (CMIA) on an Architect i2000SR analyser (Abbott Laboratories, Abbott Park, IL, USA) according to the manufacturer’s instructions (Abbot Laboratories, 2022). To compare the results with other studies, the measurement values were converted into WHO BAU/ml using a conversion factor of 0.142 (Müller et al., 2022). Values ≥ 7.1 BAU/mL were considered as positive result, according to previous validation studies (Jung et al., 2021).

### 2.4 Cellular immunity analysis

The SARS-CoV-2-specific T-cell responses were assessed by a whole-blood interferon gamma release immune assay (IGRA) using the QuantiFERON SARS-CoV-2 Research Use Only; Qiagen, Hilden, Germany. This assay consists of four tubes containing: Nil, Mitogen, SARS-CoV-2 Ag1 and Ag2. SARS-CoV-2 Ag1 and Ag2 use a combination of antigen peptides specific to SARS-CoV-2 to stimulate lymphocytes involved in cell-mediated immunity in heparinized whole blood. The SARS CoV-2 Ag1 tube contains CD4^+^ T-cell epitopes derived from the S1 subunit (receptor binding domain) of the spike protein, and the Ag2 tube contains CD4^+^ and CD8^+^ T-cell epitopes from the S1 and S2 subunits of the S protein. In conjunction with these tubes, blood containers that consist of nil and mitogen tests are used as negative and positive controls.

The concentration of IFN-γ was measured using the sandwich CLIA platform approved for the determination of cellular immunity against Mycobacterium tuberculosis-specific antigens (QuantiFERON-TB Gold Plus, Liaison XL) (DiaSorin, 2019). To calculate the final results, the nil control test needs to be subtracted from mitogen, Ag1, and Ag2 results. The CLIA platform determines IFN- γ concentrations in international units per milliliter (IU/ml). The final IFN- γ concentration in the mitogen control test needs to be > 0.5 IU/ml to validate the final Ag1 and Ag2 results. Positive response for SARS-CoV-2 Ag1 and Ag2 (calculated as SARS-CoV-2 Ag value–Nil value) was defined by the manufacturer as a value ≥ 0.3 IU/ml.

### 2.5 Statistical analyses

Statistical analyses were done using GraphPad Prism v.5.0 software. Bonferroni’s Multiple Comparison Test was used to compare the immune response between different vaccine combinations in patients without previous infection. In order to investigate the influence of previous infection on the immune response regardless of the vaccine combination, Unpaired t Test was applied. The level of significance for all tests was set up at 5%. The statistical analysis was only done among those groups with at least 7 individuals and whose immunity values ​​were within the observed range.

## 3 Results

### 3.1 Characteristics of the group of study

**Figure 1:**
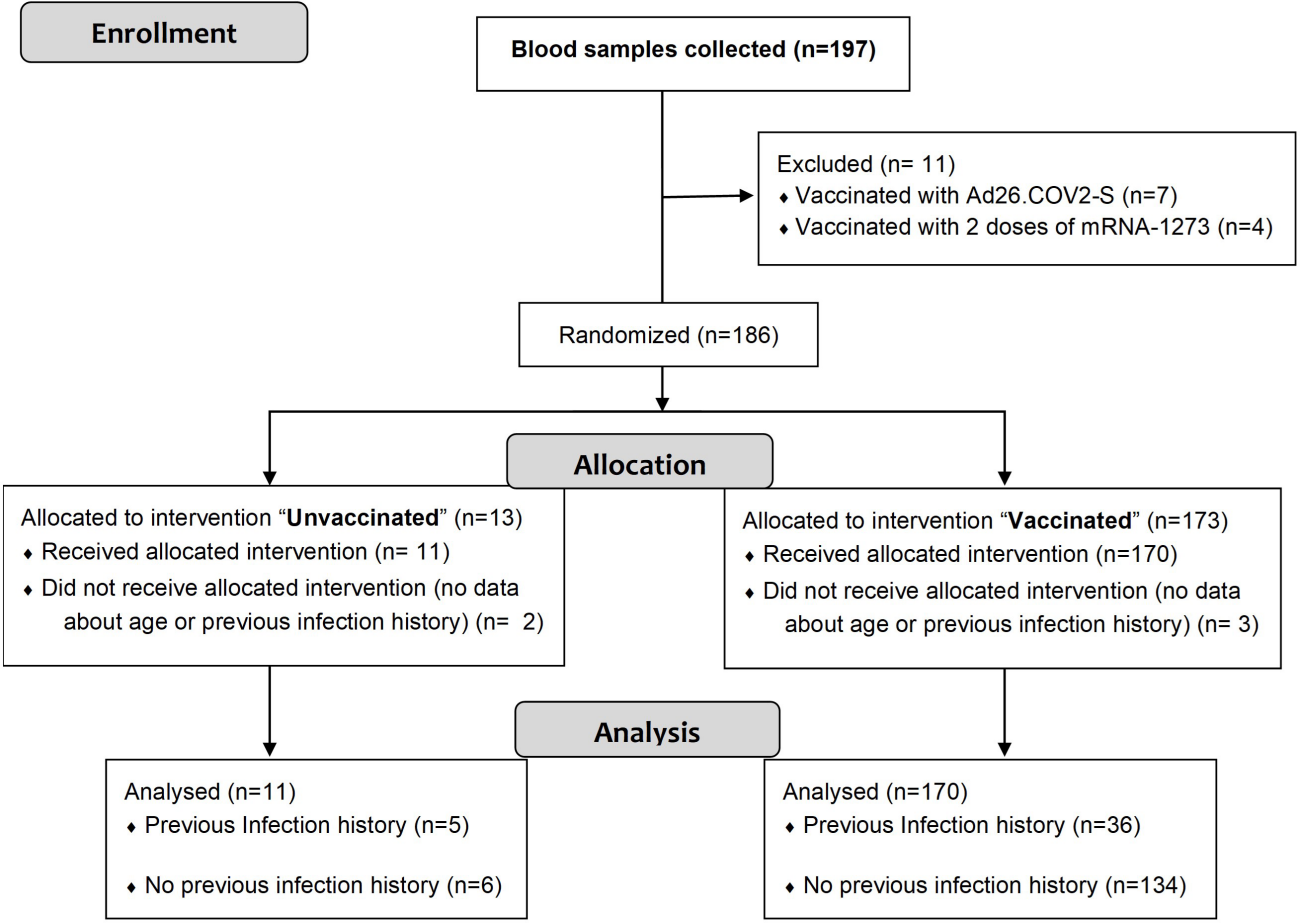
Flow diagram showing the assessment of blood samples collected.

A total of 197 blood samples were collected from consented individuals. **Figure 1** shows the flow diagram of the study procedure, including the reasons for exclusion of 16 blood samples. After assessing eligibility, 181 samples were included in the study. Of these, 170 were from vaccinated individuals having received one or two doses of the following vaccines against SARS-CoV-2: ChAdOx1-S and/or BNT162b2. Subjects immunized with a third-booster dose received BNT162b2 or mRNA-1273. The remaining 11 samples were recovered from unvaccinated participants. Overall, 41 blood samples were taken from individuals with previous infection history (5 unvaccinated and 36 vaccinated).

The baseline characteristics and the immunization (vaccination regimen and previous infection) of the participants against SARS-CoV-2 are shown in **Table 1**. A total of 162 individuals were recruited: 144 with only one blood sample, 17 patients with 2 samples and 1 patient with 3 samples in different immunological status. Among the 164 individuals, 162 were healthy people and 2 immunocompromised individuals. The age range was 24-83 years. Of the participants, 81 (61%) were women and 63 were men (39%).

**Table 1:** Baseline characteristics of study participants.

Characteristic	No. of individuals (N=162)	No. of samples (N=181)	Vaccine Regimen							
			Unvaccinated (N=11)		1 dose (N= 4)		2 doses (N= 128)		3 doses (N= 38)	
			NIH (6)	IH (5)	NIH (0)	IH (4)	NIH (109)	IH (19)	NIH (25)	IH (13)
Age (years) 24-40 41-60 > 60	41 84 37	46 101 34	0 5 1	3 2 0	0 0 0	1 3 0	32 58 19	5 11 3	3 15 7	2 7 4
Sex Male Female	63 81	79 102	2 4	4 1	0 0	2 2	59 50	3 16	7 18	2 11
Time between the last dose to blood draw (months)*	-	-	-	-	-	7 (6-8)	5.89 (2-11)	8.58 (3-12)	4.36 (1-7)	4.5 (1-7)
Immunosuppression	2	4	0	0	0	0	2	0	1	1

IH, Infection History; NIH, No Infection History. *Data are expressed as mean (maximum-minimum).

### 3.2 Immunocompromised participants

Four samples were collected from 2 Immunocompromised individuals, two of them came from a leukemia patient and the other two remaining were taken from a unique individual receiving immunosuppressant treatment (**Table 2**). The administration of two or three doses of PZ in Immunocompromised patients who had not previously been infected by SARS-CoV-2 induced a negative humoral and cellular response (< 7.1 BAU/ml and <0.3 IU/ml, respectively). However, a third dose and previous infection managed to increase the antibody level to 813.2 BAU/ml, although the cellular response remained negative.

**Table 2:** Immune response in immunocompromised individuals.

Immunosuppression	No.	Vaccine Schedule	Prior Infection	IgG-Spike Antibodies Level (BAU/ml)	CD4^+^ Response Level (IU/ml)	CD4^+^ + CD8^+^ Response Level (IU/ml)
Leukemia	Sample 1	PZ/PZ	NO	0.128	0.0425	0.188
	Sample 2	PZ/PZ/PZ	NO	0.128	0.052	0.0541
Immunosuppressant treatment	Sample 1	PZ/PZ	NO	3.962	0.0212	0.0246
	Sample 2	PZ/PZ/PZ	YES	813.220	0.0396	0.0681

### 3.3 Healthy participants

The groups studied and the median levels of the humoral and cellular immune responses are shown in **Table 3**. There were three main groups of individuals: unvaccinated, vaccinated with a primary 2-dose (AZ or PZ), and immunized with a mRNA vaccine booster (MD or PZ).

**Table 3:** Median levels of the humoral and cellular immune responses in groups of interest studied.

Immunological Status	No.	IgG-Spike Antibodies Median Level [IQR]	CD4^+^ Response Median Level [IQR]	CD4^+^ + CD8^+^ Response Median Level [IQR]
Control Groups				
Uninfected and Unvaccinated Previous Infection and no vaccination	6 5	0.1775 [0.266]	0.047 [0.109]	0.037 [0.109]
		11.417 [5.410]	0.279 [0.258]	0.405 [0.506]
Vaccinated individuals				
**Previous infection** and 1 dose of AZ	4	2182.55 [2895.29]	0.972 [0.709]	2.23 [1.515]
No infection and 2 doses of AZ	67	52.753 [68.465]	0.167 [0.194]	0.212 [0.408]
**Previous infection** and 2 doses of AZ	4	4150.085 [3987.55]	0.12 [0.299]	0.286 [0.370]
No infection and 2 doses (AZ/PZ)	7	466.640 [664.283]	0.548 [2.352]	1.07 [3.98]
**Previous Infection** and 2 doses (AZ/PZ)	1	779.154	2.73	3.03
No infection and 2 doses of PZ	33	79.108 [82.332]	0.207 [0.446]	0.278 [0.467]
**Previous infection** and 2 doses of PZ	14	162.661 [496.133]	0.5335 [0.557]	0.265 [1.032]
No infection and 3 doses (AZ/AZ/PZ)	1	2349.120	2.07	3.69
**Previous infection** and 3 doses (AZ/AZ/PZ)	1	4380.459	2.15	4.02
No infection and 3 doses (AZ/AZ/MD)	11	625.183 [1145.308]	0.638 [0.692]	0.699 [1.011]
**Previous infection** and 3 doses (AZ/AZ/MD)	2	2256.756 [1161.013]	1.045 [0.035]	1.735 [0.605]
No infection and 3 doses (PZ/PZ/PZ)	2	369.995 [279.087]	0.53 [0.351]	1.278 [1.062]
**Previous infection** and 3 doses (PZ/PZ/PZ)	3	3390.818 [1294.124]	0.814 [0.477]	1.47 [0.934]
No infection and 3 doses (PZ/PZ/MD)	10	2366.288 [1395.271]	0.557 [0.757]	1.625 [0.991]
**Previous infection** and 3 doses (PZ/PZ/MD)	6	2394.511 [1516.962]	1.165 [2.403]	2.94 [4.711]

The highest antibody titer (5672.8 BAU/ml) and T cell response (>10 IU/ml) were detected in an individual 3 months after receiving AZ/AZ vaccination and having been previously infected by SARS-CoV-2.

Participants who were not vaccinated and without knowledge of previous SARS-CoV-2 infection, developed a negative humoral response with values ​​below 2.8 BAU/ml. The infection in unvaccinated individuals contributed to a greater production of antibodies, and IFN- γ (up to 10 times) than in those without prior infection.

Even though the groups of vaccinated participants were very heterogeneous in terms of the number, in general, vaccination considerably increased the antibody titer compared to unvaccinated people. In addition, an activation of T cells producing IFN- γ was seen. All vaccinees developed a positive humoral response (>7.1 BAU/ml), but the cellular response varied depending on the vaccination regimen. In case of homologous vaccination with two doses (AZ/AZ and PZ/PZ), median T-cell response values were negative except CD4^+^ response in individuals vaccinated with double dose and previous infection. AZ/PZ combination or 3 doses of vaccination elicited a positive cellular response (median concentration of IFN- γ > 0.3 IU/ml). The level of response produced by CD4^+^ was lower than that produced by CD4^+^ + CD8^+^ T-cells in all vaccination regimen, except response induced by 2 doses of PZ and prior infection.

It is important to highlight that both humoral and cellular response following natural infection and a single dose of AZ vaccination was higher (up to approximately a 10-fold increase of the median of IFN-γ values) than those immune responses in participants with a double dose and without infection (**Table 3**).

Three patients vaccinated with two doses of AZ and without knowledge of previous SARS-CoV-2 infection, had immune response values that were outside the ranges of the rest of the group. In these three cases, anti-N IgG and IgM antibodies were tested to verify past SARS-CoV-2 infection. All of them had a negative anti-N immunity (antibodies values < 0.0142 BAU/ml). Results are shown in **Table 4**.

**Table 4:** Anti-N immunity in individuals who received double dose of AZ and without knowledge of previous SARS-CoV-2 infection.

Sample No.	IgG Antibodies (BAU/ml)	IgM Antibodies (BAU/ml)
13	0.0142	0.0369
20	0,0142	0.0369
29	0.0028	0.0185

Generally, previous infection in all vaccinated individuals contributed to enhance the median of anti-S antibodies titer. Nevertheless, the increase in the median concentration of IFN-γ by T cells was greater in AZ/PZ and 3 doses vaccinations than in the rest of vaccination regimens.

**Figure 2:**
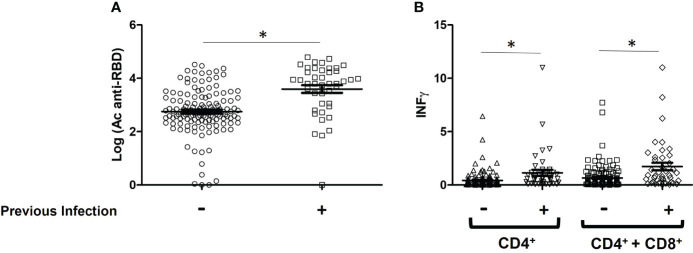
Immune response levels of vaccinated patients with and without SARS-CoV-2 infection. **(A)** Comparison of the mean values of IgG-Spike antibodies, data are expressed in BAU/ml; **(B)** IFN- γ produced by CD4^+^ and CD4^+^ + CD8^+^ T-cells, data are expressed in IU/ml. *P < 0.05.

Regardless of the type, number of doses and combination of vaccines, the influence of previous SARS-CoV-2 infection in vaccinated individuals was statistically evaluated (**Figure 2**). Overall, 36 participants were SARS-CoV-2 experienced and 134 were naive before vaccination. The mean antibody values were significantly higher in the group with previous infection compared to the group without infection (0.511 ± 0.021 vs. 0.390 ± 0.010 BAU/ml, P< 0.0001). In the case of the cellular response, statistically significant differences were also observed both in the level of IFN- γ produced by CD4^+^ (1.138 ± 0.2894 vs. 0.4296 ± 0.06573 IU/ml, P= 0.0005) and by CD4^+^ + CD8^+^ (1.727 ± 0.3518 vs. 0.6545 ± 0.09034 IU/ml, P < 0.0001).

**Figure 3:**
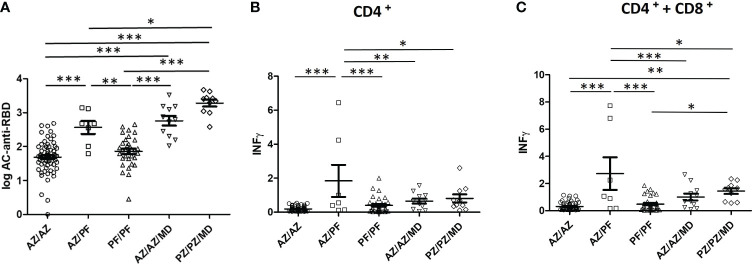
Humoral and cellular immunity elicited by several type, number, and combinations of COVID-19 vaccines. **(A)** Comparison of the mean values of IgG-Spike antibodies, data are expressed in BAU/ml; **(B, C)** IFN- γ produced by CD4^+^ and CD4^+^ + CD8^+^ T-cells, data are expressed in IU/ml *P < 0.05; **P < 0.01; ***P < 0.001.

In order to evaluate which vaccine combination elicited the highest level of immune response in individuals without previous infection, significant differences between the groups were studied. **Figure 3** shows the comparison of mean anti-Spike IgG and IFN- γ values between vaccinated groups composed of at least 7 individuals.

Considering the two-dose vaccine regimen, the highest levels of humoral and cellular immune response detected were for the AZ/PZ combination. Furthermore, the mean values were significantly increased with respect to those produced by the other two vaccine combinations that produced similar responses (AZ/AZ and PF/PF).

Taking into account the three-dose vaccine regimen, the PZ/PZ/MD combination elicited a higher concentration of anti-Spike IgG and IFN- γ than the AZ/AZ/MD combination. However, the differences were not statistically significant.

The administration of three doses significantly enhanced the production of IgG-Spike antibodies with respect to that obtained in individuals immunized with two doses of any vaccine, except AZ/PF vs AZ/AZ/MD. In case of the response of CD4^+^ and CD8^+^ T-cells, IFN-γ levels were also increased but they never exceeded those induced by the AZ/PZ combination. Statistical Analysis was varied depending on which combinations were compared. The differences in the IFN-γ concentrations were statistically significant between AZ/PZ combination and 3 doses of vaccine (AZ/AZ/MD or PZ/PZ/MD).

## 4 Discussion

In the present study, we evaluated post-COVID-19 and/or post-vaccination immunization according to the type of vaccine, number of doses and combination of vaccines. The IgG antibodies against the S protein and IFN-γ- producing CD4^+^ and CD8^+^ T lymphocytes are a reliable read-out of humoral and cellular immunity to SARS-CoV-2 following natural infection and vaccination.

Our results revealed that previous exposure to SARS-CoV-2 antigens by infection strongly affected subsequent cellular and humoral immunity. In fact, among the unvaccinated participants, infected people managed to develop IFN- γ levels up to 10 times higher than those without prior infection. The data from vaccinated individuals demonstrated that humoral and cellular immune responses were significantly higher in SARS-CoV-2 experienced compared to naive participants, regardless of the type, number of doses and combination of vaccines. Our observations are consistent with previous studies showing that history of SARS-CoV-2 infection influences the increase in immunity of vaccinated people (Angyal et al., 2021; Tretyn et al., 2021; Desmecht et al., 2022).

Among healthy people, the level of protection against COVID-19 could be enhanced with vaccination, reaching antibodies and IFN- γ values similar or even higher than those detected in COVID-19 patients (Grigoryan and Pulendran, 2020; Kedzierska and Thomas, 2022). Based on our results, high antibody titer was observed in all vaccinated individuals compared to titer from unvaccinated participants. In addition to these effects on the antibody response, CD4^+^ and CD8^+^ T cell responses were also induced following vaccination. As described Grigoryan et al., the mRNA and viral-vector-based platforms may be beneficial for the induction of T cell responses (Grigoryan and Pulendran, 2020). Given that Ag1 stimulates production of IFN- γ by CD4^+^ T cells and Ag2 stimulates both CD4^+^ and CD8^+^ T cells, mainly median concentration of IFN- γ by both cell subpopulations was greater than that of CD4^+^ alone. Nevertheless, several authors have shown that IFN-γ by CD4^+^ T cells numerically dominated over IFN- γ by CD8^+^ T cells after COVID-19 immunization (Murugesan et al., 2021; Barreiro et al., 2022), similar to what was found in our study for individuals vaccinated with 2 doses of PZ and previous infection. Moreover, Matew et al (Mathew et al., 2020) observed that CD8^+^ T cells are not present in all infected patients and ~20% of patients did not have substantial CD8^+^ T-cell activation. Hence, it could be possible that in the present study, those individuals with a prior infection before receiving 2 doses of PZ would not have had a CD8^+^ T-cell activation.

Conversely, patients with acquired or inherited immune disorders may show variable immune responses to vaccination against SARS-CoV-2 (Oyaert et al., 2022; Fiorcari et al., 2023). In the present study, the two immunocompromised participants developed a negative humoral and cellular response after 8 months and 1 month after administration of the second and third dose, respectively. These results are in line with Oyaert et al. (2022) who evaluated the humoral and cellular immune response in a large cohort of patients diagnosed with immune deficiency disorders and revealed incomplete and/or delayed humoral and cellular immune responses up to 3 months after administration of the second dose. However, the participant who had been infected between the first and second dose, had positive IgG anti-S antibodies at 2 months after administration of the third dose, confirming that prior SARS-CoV-2 infection improves immune response.

One vaccine dose could be sufficient to increase both cellular and humoral immune response in COVID-19–recovered subjects (Angyal et al., 2021; Ebinger et al., 2021; Mazzoni et al., 2021; Tretyn et al., 2021; Varghese et al., 2022), even surpassing the titer achieved by the second dose of vaccine in SARS CoV-2 naive recipients (Varghese et al., 2022). These findings were obtained in the present study, the immune response after the first dose of the AZ vaccine in previously infected people was higher than after two doses of AZ in those who had never had the disease. A prior SARS-CoV-2 infection triggered the immune system to a very strong response to a single dose of the vaccine. The first dose, given in to people whose immune systems had already been stimulated by the natural infection, had a similar effect when given as a second “booster” dose (Tretyn et al., 2021).

Our data is part of two projects in which the immune response to several SARS-CoV-2 vaccines was evaluated. The results of these studies demonstrated that after vaccine inmunization, those people without previous infection developed a level of anti-N IgG and IgM antibodies values of 0.00142-0.0071 and 0.00142-0.0142 BAU/ml, respectively. These antibody titers were lower than those found in the three patients vaccinated with two doses of AZ and without knowledge of previous infection, whose immune response (anti-S antibodies and IFN- γ produced by T-cell) values were outside the ranges of the rest of the group. Therefore, despite anti-N antibody titers of < 0.142 BAU/ml, probably these three patients could have had asymptomatic SARS-CoV-2 infection at some point prior to analysis. Our observations are in line with previous studies showing a higher concentration of IFN- γ produced by T cells in asymptomatic than in symptomatic patients (Le Bert et al., 2021; Zhao et al., 2021).

Regarding a two-dose vaccination regimen, the highest humoral and cellular immunity detected was for AZ/PZ combination. Several reports have published that the mixing of the AZ and PZ vaccine induce a more robust immune response compared to that induced by the two doses of either of these vaccines (Borobia et al., 2021; Schmidt et al., 2021). Besides, our results demonstrated that the cellular immune response to the heterologous mix was even significantly greater than that to AZ/AZ/AZ and PZ/PZ/PZ combinations.

On the other hand, a third boosting vaccine dose not only reduces the risk of symptomatic breakthrough infections by new SARS-CoV-2 variants (Zhang B. et al., 2022) but also the incidence of severe cases and mortality related to COVID-19 (Muhsen et al., 2021), particularly in uninfected individuals. Moreover, both humoral and cellular immunity waned within 6 months after the first two vaccine doses, highlighting the urgency of administering a booster dose to induce stronger immunity against SARS-CoV-2 (Tartof et al., 2021; Uwamino et al., 2022). In addition, the benefit of the booster dose is greater in native individuals who have lower persisting levels of humoral or cellular immunity before the booster dose (Desmecht et al., 2022). Based on both our results and previous study published by Demecht et al, the booster dose induced an increase in humoral and cellular immune responses (Desmecht et al., 2022). Based on the concentration of anti-Spike antibodies, we found that individuals after receiving a third boosting vaccine dose (MD) had significantly higher antibody titers than participants who had received two doses of homologous vaccination (PZ/PZ or AZ/AZ). These findings are consistent with previous study reporting that mRNA Booster Vaccination (PZ or MD) Enhanced Antibody Response against SARS-CoV-2 in individuals vaccinated with either two prior doses of mRNA (PZ) or inactivated virus vaccine (AZ) (Zhang B. et al., 2022).

One of the limitations of our study was the heterogeneity in terms of the number of participants in each group studied, since the sample size was not calculated at the beginning of the study. This is due to the random inclusion of subjects, the participants were recruited as they voluntarily provided consent and as the reagents became available to perform the assays.

In summary, the present study demonstrate that heterologous vaccination (AZ/PZ) induced the strongest immunity among the two-dose vaccination regimens. The immunity offered by the third-booster dose of SARS-CoV-2 vaccine depends not only on the type of vaccine administered but also on previous doses and prior infection. Previous exposure to SARS-CoV-2 antigens by infection strongly affect immunity of vaccinated individuals.

## Data availability statement

The raw data supporting the conclusions of this article will be made available by the authors, without undue reservation.

## Ethics statement

This study involving humans was approved by Ethics Committee of Hospital Clınico San Carlos, Madrid (Spain) (References: 21/071-E and 21/ ´193-E). All research was conducted according to the Declaration of Helsinki principles. The participants provided their written informed consent to participate in this study.

## Author contributions

EC: Supervision, Conceptualization, Writing – review & editing, Writing – original draft, Visualization, Validation, Investigation, Data curation. MM: Writing – review & editing, Writing – original draft, Methodology, Investigation. CN: Methodology, Investigation, Writing – review & editing, Writing – original draft. JL: Writing – review & editing, Writing – original draft, Methodology, Investigation. EM: Writing – review & editing, Writing – original draft, Methodology, Investigation. AD-I: Validation, Supervision, Conceptualization, Writing – review & editing, Writing – original draft. ER: Visualization, Formal analysis, Data curation, Writing – review & editing, Writing – original draft, Validation.
